# Diagnostic Performance Between Chest CT Severity Score and Initial Reverse Transcription-Polymerase Chain Reaction (RT-PCR) Cycle Values in COVID-19 Patients and Their Relation With the Clinical Status of Patients

**DOI:** 10.7759/cureus.47733

**Published:** 2023-10-26

**Authors:** Ishan Banerjee, Vivek Sullere, Manish Jain, Koushik Biswas

**Affiliations:** 1 General Medicine, Bombay Hospital, Indore, IND; 2 Cardiology, Bombay Hospital, Indore, IND; 3 Biochemistry, All India Institute of Medical Sciences, Raebareli, IND

**Keywords:** ct-ss, covid-19 pneumonia, hrct thorax, ctss, ct value, rt-pct, covid-19, sars-cov-2

## Abstract

Introduction: Reverse transcription-polymerase chain reaction (RT-PCR) is used as a standard test for the diagnosis of SARS-CoV-2 viral RNA from nasopharyngeal aspirates. However, this method lacks sensitivity and cannot assess disease severity. A CT scan of the thorax provides a CT severity score (CT-SS), which depicts lung involvement and disease severity. This study aims to investigate the diagnostic value of chest CT compared with RT-PCR cycle threshold (Ct) values in COVID-19 and relate it clinically with the disease severity of patients.

Methods: This retrospective observational study was conducted in a tertiary center from April 2021 to March 2022. We included 511 patients who had tested RT-PCR positive for COVID-19, were hospitalized, and had undergone high-resolution CT (HRCT) thorax. Data was collected from patient records regarding name, age, sex, admission data, baseline investigations including Ct value, management, and outcome. HRCT was reviewed to assess lung involvement and calculate CT-SS. Data was analyzed using SPSS Statistics version 25 (IBM Corp. Released 2017. IBM SPSS Statistics for Windows, Version 25.0. Armonk, NY: IBM Corp.).

Result: The mean age of patients was 50.4 ± 13.7 years, and the majority (67.5%) were male. Gender-wise, there was no difference in RT-PCR cycle threshold (Ct) values; however, CT-SS was significantly higher in males (17.5 ± 4.8 vs.10.5 ± 6.6, t=-13.6, p<0.0001). ICU admission was needed for 34.8% of patients, and they had a significantly lower Ct value (21.7 ± 3.3 vs. 22.8 ± 3.7, t=21.10, p<0.0001) and higher CT-SS (16.3 ± 4.5 vs. 6.7 ± 5.1, t=-3.32, p=0.001).

Conclusion: Ct values could not differentiate between moderate and severe patients. CT-SS was not related to the viral load at admission. Patients who succumbed had significantly lower Ct values and higher CT-SS.

## Introduction

The first case of COVID-19 was detected in Wuhan, China, in December 2019. Thereafter, it has spread across the world, resulting in a pandemic. COVID-19 cases have been diagnosed to be caused by SARS-CoV-2 [1]. The SARS-CoV-2 is an enveloped, pleomorphic, positive-sense, single-stranded RNA virus [2]. Most people infected with the virus will experience mild to moderate respiratory illness and recover without requiring special treatment. However, some will become seriously ill and require medical attention. Conditions like acute respiratory distress syndrome, septic shock, acute kidney injury, cardiac injury, and multi-organ failure are reported as complications of COVID-19 infection. Patients with advanced age, hypertension, diabetes mellitus, cardiovascular diseases, and chronic lung disease are at higher risk of developing complications from COVID-19. Hence, early diagnosis is critical for classifying patients based on the severity of illness to improve their prognosis [2,3].

COVID-19 virus can be detected by molecular methods, serology, and viral culture. The sensitivity of the diagnostic test mainly depends on the stage of the illness when the sample is collected. During the first week of infection, when there are insufficient copies of viral RNA, proteins, or virus-specific antibodies in circulation, serological and molecular tests are not useful [4]. Hence, the chances of determining the presence of the virus remain low before the onset of symptoms. Viral culture is a more time-consuming method compared to the other methods. Culture is much more useful in the first stage of an outbreak before other diagnostic methods become clinically available [5]. The most common diagnostic methods used are molecular methods such as reverse transcription-polymerase chain reaction (RT-PCR) test or real-time polymerase chain reaction (PCR) test, which identifies viral RNA in respiratory samples such as nasopharyngeal aspirate, pharyngeal swabs, deep tracheal aspirate, sputum, or bronchoalveolar lavage [6].

Despite the knowledge of the common signs and symptoms like fever, dry cough, fatigue, dyspnea, anorexia, and myalgia, clinical diagnosis is challenging owing to the overlapping of symptoms with other viral pneumonia-like influenza A and B and gastroenteritis. A single RT-PCR is reported to be less sensitive owing to issues like improper sampling, low patient viral load, sample collection time, or laboratory factors [7]. In our series, the sensitivity of chest CT was greater than that of RT-PCR (98% vs. 71%, respectively; p<0.001) [8]. Moreover, there are chances of false negative results in RT-PCR tests if the specimen gets contaminated with amplification inhibitors or if the specimen contains an inadequate quantity of viral particles [9,10]. A CT scan is helpful in such a situation as it can detect early parenchymal involvement and disease progression related to COVID-19 infection and also provides alternative diagnoses. A chest CT scan provides a CT severity score (CT-SS), a semi-quantitative scoring system determining the extent and severity of pulmonary involvement in viral pneumonia. A CT scan is reported to have a high sensitivity (97%) but poor specificity (25%) when compared to RT-PCR for COVID-19 diagnosis [11]. This is ascribed to the fact that the CT appearance in COVID-19 overlaps with the appearance of other viral pneumonia, similar to influenza viruses, para influenza virus, respiratory syncytial virus, adenovirus, human metapneumovirus, rhinovirus, etc. [12-14].

RT-PCR tests are usually reported qualitatively as positive or negative using a specified cut-off, either based on cycle threshold (Ct) or integrated by an automatic algorithm that interprets different parameters of potential amplification. Ct values are not reported in normal circumstances. RT-PCR Ct values denote the number of amplification cycles required for the target gene to exceed a threshold level. Ct values are hence inversely proportional to the viral load and provide an indirect estimate of the copy number of viral RNA in the patient’s sample [15].

Whether SARS-CoV-2 Ct values can help in guiding patient management decisions is unknown; we need to understand and gather further evidence if SARS-CoV-2 Ct values correlate with clinical outcomes and, therefore, whether they could provide valuable information to clinicians for more tailored decision-making [15]. In this study, we reported the results of chest CT compared to the initial RT-PCR results in patients with confirmed diagnosis of COVID-19. This study aims to investigate the diagnostic value of chest CT compared with RT-PCR cycle values in COVID-19 and to relate it clinically with the disease severity of patients.

## Materials and methods

Study design and participants

This hospital-based retrospective observational study was conducted among COVID-19 RT-PCR/antigen/antibody test-positive patients referred to the Department of General Medicine, Bombay Hospital, Indore, India. The study was conducted from April 2021 to March 2022 for one year.

Data collection

A predesigned, self-administered proforma was designed to record the history, examination details, and investigation reports of patients systematically, keeping the objectives of the study at the center point. Patients were selected for the study based on the inclusion and exclusion criteria. A data collection sheet was used to collect and record the detailed history, including name, age, sex, admission data, baseline blood investigations, RT-PCR at admission, and other test parameters.

The high-resolution CT (HRCT) chest was conducted on a Siemens Somatom Definition AS 64 slice CT scan machine (Siemens Medical Solutions USA, Inc., Pennsylvania, USA). Routine axial, coronal, and sagittal sections were included. No intravenous contrast medium was administered. All CT images were reviewed and interpreted independently by trained radiologists in our hospital. We also interpreted the chest CT of each patient for CT-SS. The grading of each lung lobe was as follows: Grade 0 (no involvement), Grade 1 (<5% involvement), Grade 2 (5-25% involvement), Grade 3 (26-49% involvement), Grade 4 (50-75% involvement), and Grade 5 (>75% involvement).

The total score of the five lung lobes was the lesion score of each patient. The score ranged from 0 (no involvement) to 25 (maximum involvement) [16]. A total score of 1-8 was classified as mild, 9-15 as moderate, and 16-25 as severe.

The Ct values of the study patients who had undergone RT-PCR (TaqPath COVID‐19 Combo Kit, Applied Biosystems, Massachusetts, USA) with nasopharyngeal swabs were categorized as high load (Ct 1-25), moderate load (Ct 26-30), and mild load (Ct >30), while a Ct value over 40 was deemed as negative for COVID-19 [17].

After collecting data using the predesigned proforma, data variables were compiled in an Excel spreadsheet (Microsoft Corporation, Washington, USA).

The inclusion criteria for this study include patients of both sexes aged over 18 years, who have tested positive on RT-PCR, antigen, or Antibody tests, and have undergone CT scan evaluation.

The exclusion criteria for this study include patients with physical disabilities, COVID-19-positive individuals engaged in occupations with a high risk of occupational lung disease, such as coal workers, asbestos factory employees, farmers, and silica miners, and patients who have passed away before undergoing a CT scan examination.

We calculated the sample size based on the following formula:

SS = (Z-score)² * p*(1-p) / (e)²

where SS is the sample size, Z-score is the critical value and the standard value for the corresponding level of confidence (1.96 for confidence level 95%), p is the pre-determined value of sensitivity (or specificity) determined from previous published data or clinician experience/judgment, and e is the margin of error (margin of error of 5% is taken) SS = (1.96)2 * 0.70 * (1-0.70) / (0.05)2

Considering the average sensitivity of the SARS-CoV-2 RT-PCR test to be 70% [18], the sample size was 322.69. Hence, the minimum sample size for the study was 323 patients. We included 511 patients during our study period based on the inclusion and exclusion criteria.

Ethical approval

Ethical approval was taken from the Institute Ethical Committee and Institute Scientific Committee of Bombay Hospital, Indore, India, before starting the study (BHI: DDMS: EC: 2021-03:008).

Data management and statistical analysis

Strict confidentiality was maintained throughout the study regarding the patient data utilized for the current study. All the categorical data were presented as frequency and percentage. The continuous data was checked for normality using the Kolmogorov-Smirnov test. The parametric data was presented as mean ± standard deviations, while the non-parametric data was presented as median and interquartile range. An independent sample t-test was used to calculate the significance of study parameters within two groups for parametric data. A one-way ANOVA test was used to calculate the significance of study parameters between three groups for parametric data. A p-value of <0.05 was considered as a level of significance. Data was analyzed using SPSS Statistics version 25 (IBM Corp. Released 2017. IBM SPSS Statistics for Windows, Version 25.0. Armonk, NY: IBM Corp.).

## Results

In our study, 345 patients (67.5%) were males and 166 patients (32.5%) were females. The mean age of male and female patients in the study was 50.7 ± 13.4 years and 49.9 ± 14.4 years, respectively. The Ct value and CT-SS score of male and female patients is given in Table 1.

**Table 1:** Gender-wise Ct value and CT-SS score Ct value: RT-PCR cycle value, CT-SS: CT severity score, SD: standard deviation

	Male (n=345)	Female (n=166)	t-value	p-value
Age (mean ± SD)	50.7 ± 13.4	49.9 ± 14.4	-0.617	0.5377
Ct value (mean ± SD)	22.2 ± 3.4	21.9 ± 3.6	-0.916	0.3599
CT-SS score (mean ± SD)	17.5 ± 4.8	10.5 ± 6.6	-13.6	<0.0001

We subdivided the patients based on age and estimated the Ct value and CT-SS score for patients of each age group. A one-way ANOVA did not reveal any significant statistical difference between Ct value (F=0.353, p=0.842) and CT-SS (F=1.899, p=0.109) between patients of different age groups (Table 2).

**Table 2:** Age group distribution and their respective Ct value and CT-SS Ct value: RT-PCR cycle value, CT-SS: CT severity score, SD: standard deviation, ANOVA: analysis of variance

Age group	18-29 years	30-39 years	40-49 years	50-59 years	≥ 60 years	One-way ANOVA: p-value
n (%)	44 (8.7)	80 (15.7)	97 (18.9)	142 (27.8)	148 (28.9)	
Age in years (mean ± SD)	24.9 ±3.3	34.9±2.7	44.8 ± 2.9	54.9 ± 2.9	65.7 ± 5.3	
Ct value (mean ± SD)	21.9 ± 3.9	22.1±3.6	22.4 ± 3.1	21.9 ± 3.5	22.1 ± 3.2	0.353; 0.842
CT-SS (mean ± SD)	8.4 ± 6.6	10.9±6.3	10.4 ± 7.3	9.5 ± 6.8	10.4 ± 6.2	1.899; 0.109

Of 511 COVID-19 patients in our study, 333 patients (65.2%) were admitted to the general medicine ward, and 178 patients (34.8%) were admitted to the ICU. The Ct value of patients admitted to the ICU was significantly lower than those admitted to the ward (21.7 ± 3.3 vs. 22.8 ± 3.7, p<0.0001). The CT-SS of the patients admitted to the ICU was significantly higher than those admitted to the ward (16.3 ± 4.5 vs. 6.7 ± 5.1, p=0.001). The CT value and CT-SS of these two groups of patients are presented in Table 3.

**Table 3:** Ct value and CT-SS score of patients based on admission ward ICU: intensive care unit, Ct value: RT-PCR cycle value, CT-SS: CT severity score, SD: standard deviation

Admission ward	General ward (n=333)	ICU (n=178)	t-value	p-value
Ct value (mean ± SD)	22.8 ± 3.7	21.7 ± 3.3	21.10	<0.0001
CT-SS (mean ± SD)	6.7 ± 5.1	16.3 ± 4.5	-3.322	0.0010

Different types of oxygen support were provided to COVID-19 patients in our study. The CT value and CT-SS of patients requiring different types of oxygen support are presented in Table 4.

**Table 4:** Need of oxygen support and their respective Ct value and CT-SS score O_2_: oxygen, HFNO: high flow nasal oxygen, Bi-PAP: bilevel positive airflow pressure, Ct value: RT-PCR cycle value, CT-SS: CT severity score, SD: standard deviation

Oxygen support	Intranasal O_2 _(n=306)	HFNO (n=77)	Bi-PAP (n=58)	Ventilator (n=70)
Ct value (mean ± SD)	20.5 ± 3.2	21.4 ± 3.6	21.7 ± 2.8	24.2 ± 4.2
CT-SS score (mean ± SD)	9.7 ± 5.4	11.8 ± 3.9	12.7 ± 2.8	16.8 ± 4.7

Depending on the CT-SS of patients included in our study, 41.5% had a mild infection, 33.8% had a moderate infection, and 24.7% had a severe infection. The Ct value and CT-SS of these three groups are presented in Table 5.

**Table 5:** Distribution of patients based on their CT-SS severity Ct value: RT-PCR cycle value, CT-SS: CT severity score, SD: standard deviation, ANOVA: analysis of variance

Severity (CT-SS)	Mild (1-8) (I) (n=212)	Moderate (9-15) (II) (n=173)	Severe (16-25) (III) (n=126)	ANOVA: p-value	Tukey's posthoc analysis
Ct value (mean ± SD)	22.8 ± 2.8	21.4 ± 3.1	22.0 ± 4.6	6.331; 0.002	I>II=III

Based on RT-PCR taken at the time of admission, 11.1% of patients had a moderate viral load, and 89.9% had a high viral load. The mean CT value and CT-SS score of these two groups of patients are presented in Table 6.

**Table 6:** Distribution of patients based on their viral load detected using RT-PCR Ct value: RT-PCR cycle value, CT-SS: CT severity score, SD: standard deviation

Viral load (Ct value at admission)	Moderate load (26 – 30) (n=57)	High load (1 – 25) (n=443)	t-value	p-value
CT-SS (Mean ± SD)	13.91 ± 6.3	10.61 ± 5.6	-4.13	0.001

We observed that 79.1% of patients had recovered from COVID-19 infection, while 20.9% had died during their hospital stay. Patients were considered to have recovered from COVID-19 when they did not have a fever for the last seven days, did not need any oxygen support for the last seven days, and were otherwise clinically stable. The mean Ct value and CT-SS score of these two groups are presented in Table 7.

**Table 7:** Distribution of patients based on their outcome Ct value: RT-PCR cycle value, CT-SS: CT severity score, SD: standard deviation

Outcome	Death (n=107)	Recovered (n=404)	t-value	p-value
Ct value (mean ± SD)	20.3 ± 4.1	22.1 ± 3.3	4.755	<0.0001
CT-SS (mean ± SD)	16.3 ± 4.7	8.4 ± 6.1	-12.450	<0.0001

## Discussion

In our study, we carried out a retrospective analysis of 511 SARS-CoV-2 RT-PCR test-positive hospitalized patients who had also undergone an HRCT chest to investigate the diagnostic value of chest CT compared to RT-PCR Ct values and relate it with disease severity. Early diagnosis and stratification of COVID-19 patients according to disease severity is important to initiate management and reduce mortality. Our study observed males (67.5%) preponderance compared to females (32.5%). The sex distribution of patients in our study is similar to that reported by Francone et al. [19].

We found no difference in RT-PCR Ct values between male and female patients. However, the CT-SS was significantly higher in male patients, reflecting a higher severity of COVID-19 pneumonia in male patients (Table 1). This may be attributed to the fact that as SARS-CoV-2 IgG antibody levels are higher in females than males [20], it may protect against the development of COVID-19 pneumonia. We did not observe any significant difference in the Ct value and CT-SS score among patients of different age groups (Table 2).

In our study, 65.2% (n=333) of the patients were admitted to the ward, and the rest, 34.8% (n=178), were admitted to the ICU. The Ct value of patients admitted to the ICU was significantly lower than those admitted to the ward. This may be because patients admitted to the ICU had a higher viral load at the time of admission. The CT-SS of the patients admitted to the ICU was significantly higher than those admitted to the ward (Table 3). This is likely because patients who developed COVID-19 pneumonia had a higher CT-SS and had to be managed in the ICU. The proportion of patients admitted to the ICU in our study was higher than a previous study conducted in India [21].

We observed that patients who required mechanical ventilation had higher CT-SS but not necessarily lower Ct values (p<0.05) (Table 4). This may be because Ct depends on the timing and quantity of sample collected [3]. Agarwal et al. also reported that oxygen requirement was significantly correlated with CT-SS [22]. Rajyalakshmi et al. [21] also reported that Ct cannot predict the requirement of invasive ventilation.

COVID-19 patients are classified into mild, moderate, severe, and critical based on the severity of the presentation. Mild to moderate COVID-19 is found in about 80% of patients. Despite low mortality, patients in mild to moderate stages may progress to severe or critical stages in a week [23]. Based on the CT-SS scoring system, this study divided patients into mild, moderate, and severe groups. When Ct values were compared between these groups, we observed a statistically significant difference between the mild vs. moderate and mild vs. severe groups but not between the moderate vs. severe group (ANOVA 6.331, p=0.002) (Table 5).

Based on RT-PCR Ct value, 11.1% had moderate, and 89.9% had high viral load at the time of admission (Table 6). When CT-SS were compared among these two groups, we observed that the CT-SS was significantly higher in patients with lower viral load (t=-4.13; p=0.001). Although some studies have reported an association between disease severity and Ct values, another study reported no difference in mean viral loads among COVID-19 patients with and without pneumonia [24-25].

The recovery rate in the present study was 79.1%, and the mortality rate was 20.9%. Patients who succumbed had significantly lower Ct values and higher CT-SS than those who recovered from the disease (Table 7). This may be because they had higher viral load at the time of admission and were more affected by COVID-19 pneumonia. Rajyalakshmi et al. [21] also reported that a low Ct value was associated with higher mortality. Raoufi et al. [26] had previously reported that the mean CT-SS of non-survivors was significantly higher.

In this sudden pandemic, RT-PCR revealed the cause of the outbreak. The sensitivity of antibody detection is usually lower than molecular methods, mainly used in retrospective diagnosis. However, there are some ambiguities, such as whether it is best to use Ct values in predicting the level of infectivity and disease severity in patients infected with SARS-CoV-2. We classified patients into different groups based on their RT-PCR Ct value at the time of admission and CT-SS. Then, we tried to understand the clinical course and outcome of patients in each group.

Our study was not without limitations. As this is a single-center study, the findings cannot be generalized. Owing to the limited literature on assessing the efficacy of different commercially available RT-PCR kits for pool sample testing, one should be cautious in generalizing our findings as many diagnostic kits vary in sampling method. Our patients were not subjected to repeated RT-PCR tests, limiting the overall comparison at different stages of disease severity. Other respiratory tract infections that have similar patterns in the HRCT thorax could not be assessed in our study. Future studies should be designed with larger sample sizes and different commercially available RT-PCR kits to overcome these shortcomings and develop an idea of applying the Ct value of RT-PCR.

## Conclusions

CT-SS classifies COVID-19 patients into mild, moderate, and severe groups. Our findings indicate that the RT-PCT Ct value was able to differentiate between mild vs. moderate and mild vs. severe COVID-19 pneumonia patients but could not differentiate between moderate vs. severe COVID-19 pneumonia patients. We also classified patients into moderate and high viral load groups based on the RT-PCR Ct value at admission. We observed that CT-SS was not related to the viral load at the time of admission. No comparison was observed between the Ct value and the requirement of mechanical ventilation. Although Ct values may give a rough estimate of viral load, our findings are not robust enough to support that it can help differentiate the mild, moderate, and severe forms of the disease. The patients who succumbed in our study had lower Ct values and higher CT-SS. Hence, we advocate close monitoring of such patients. Longitudinal studies with adequate sample size should further evaluate viral dynamics to better interpret Ct values by the time of testing since the onset of symptoms.
